# Oral Hygiene With Neutral Electrolyzed Water and Systemic Therapy Increases Gastric *Helicobacter pylori* Eradication and Reduces Recurrence

**DOI:** 10.1002/cre2.927

**Published:** 2024-07-07

**Authors:** Victor Hugo Urrutia‐Baca, Brenda Astrid Paz‐Michel, Alma Nidia Calderon‐Porras, Jany Ariadne Jiménez‐Del Valle, Wendy Jazmin Alvarez‐Fernández, Nicolas Mervitch‐Sigal, Mario Alfredo Rodríguez‐León, Myriam Angelica De La Garza‐Ramos

**Affiliations:** ^1^ Facultad de Ciencias Biológicas Universidad Autonoma de Nuevo Leon San Nicolas de los Garza Nuevo Leon Mexico; ^2^ Department of Research Esteripharma S.A. de C.V. Atlacomulco Estado de Mexico Mexico; ^3^ Escuela de Estomatología Universidad de Montemorelos Montemorelos Nuevo Leon Mexico; ^4^ Department of Medical Administration Esteripharma México S.A. de C.V. Mexico City Mexico; ^5^ Facultad de Odontología Universidad Autonoma de Nuevo Leon Monterrey Nuevo Leon Mexico

**Keywords:** gastric cancer, *H. pylori* infection, neutral electrolyzed water, oral hygiene

## Abstract

**Objectives:**

*Helicobacter pylori* gastric infection strongly correlates with gastric diseases such as chronic gastritis, functional dyspepsia, and complications such as peptic ulcers and gastric cancer. In developing countries, systemic therapies are not usually successful due to elevated antibiotic resistance. Additionally, oral *H. pylori* infection and periodontal disease correlate with gastric treatment failures. This study aimed to explore the effect of an integral therapy, comprising oral hygiene and concomitant systemic treatment, to increase the eradication of gastric infection and recurrences.

**Materials and Methods:**

A prospective, randomized, four‐arm, parallel‐group, open‐label clinical trial was conducted to investigate the efficacy of integral therapy to eradicate gastric *H. pylori* infection and avoid recurrences in double‐positive (real‐time PCR oral and gastric infection) patients. Oral hygiene involved mouthwash with neutral electrolyzed water (NEW), with or without periodontal treatment. One hundred patients were equally distributed into four groups: NS, NS‐PT, NEW, and NEW‐PT. All patients had concomitant systemic therapy and additionally, the following oral treatments: mouthwash with normal saline (NS), periodontal treatment and mouthwash with normal saline (NS‐PT), mouthwash with NEW (NEW), and periodontal treatment and mouthwash with NEW (NEW‐PT). Gastric and oral infection and symptoms were evaluated one and four months after treatments.

**Results:**

Integral therapy with NEW‐PT increased gastric eradication rates compared with NS or NS‐PT (84%−96% vs. 20%−56%; *p* < 0.001). Even more, a protective effect of 81.2% (RR = 0.1877; 95% CI: 0.0658−0.5355; *p* = 0.0018) against recurrences and 76.6% (RR = 0.2439; 95% CI: 0.1380−0.4310; *p* < 0.001) against treatment failure (eradication of infection and associated symptoms) was observed in patients from the NEW and NEW‐PT groups.

**Conclusions:**

Implementation of oral hygiene and systemic treatment can increase the eradication of gastric infection, associated symptoms, and recurrences. NEW is recommended as an antiseptic mouthwash due to its efficacy and short‐ and long‐term safety.

## 1 Introduction

*Helicobacter pylori*, a gram‐negative bacterium of global prevalence, survives in very low pH environments, such as human gastric mucosa. *H. pylori* infection has been associated with gastrointestinal pathologies, such as chronic gastritis and dyspepsia (Alexander, Retnakumar, and Chouhan 2021; Watari 2014). About half of the world's population is currently colonized. Infection commonly happens in childhood, and its prevalence strongly correlates with socioeconomic and hygienic conditions (Sipponen and Maaroos 2015; Valdez‐Gonzalez et al. 2014; Zamani et al. 2018). In the case of Latin America and Caribbean countries, a recent meta‐analysis revealed that 50% of the children and adolescents were colonized, while 70% of adults were infected. No significant differences were found concerning gender, and it was concluded that the prevalence of *H. pylori* is high and independent of sex and age in these world regions (Curado, de Oliveira, and de Araújo Fagundes 2019). *H. pylori* infection is transmitted orally (oral−oral, fecal−oral, and gastro−oral) and can spread by consuming contaminated food and drink. Smoking, overcrowded living (sharing food and housing habits), and periodontal disease are risk factors for infection (Kayali, Manfredi, and Gaiani 2018; Li et al. 2021; Mendoza‐Cantú et al. 2017). Despite developing chronic gastritis, nearly 80% of infected individuals are asymptomatic (Malfertheiner et al. 2023). In the remaining individuals, symptoms of chronic gastritis and dyspepsia, heartburn, nausea, vomiting, loss of appetite, bloating, belching, burning upper abdominal pain, anxiety, and depression occur (Al Quraan et al. 2019). The complications of these conditions can lead to peptic ulcers, and close to 1%–3% evolve into gastric carcinoma (Ahn and Lee 2015; Malfertheiner et al. 2023; Rawla and Barsouk 2019). *H. pylori* is responsible for around 75% of all non‐cardia cancers and 63% of all gastric cancers (Kesharwani, Dighe, and Lamture 2023). Thus, eradicating the infection with triple or quadruple therapy has been the gold standard for alleviating functional dyspepsia and decreasing the incidence of gastric carcinogenesis and peptic ulcers (Ahn and Lee 2015). Nevertheless, resistance to common antibiotics against *H. pylori* has increased worldwide, particularly in Latin America, with rates of 12% for clarithromycin, 53% for metronidazole, 4% for amoxicillin, 6% for tetracycline, 3% for furazolidone, and 15% for fluoroquinolones (Ahn and Lee 2015; Camargo et al. 2014). A recent report about the Chilean population (Morgan et al. 2023) described single‐ or multi‐antibiotic resistances as high as 61.8% or 20.7%, respectively. This loss of susceptibility developed by the bacteria is a major cause of refractory *H. pylori* gastritis and dyspepsia, and patients with refractory disease have important deterioration in their quality of life (Hanafy and Seleem 2019; Jiang et al. 2015). Additionally, *H. pylori* have been demonstrated in the oral cavity and identified as an important risk factor for gastric infection, pathology, and recurrence (Anand, Kamath, and Anil 2014; Flores‐Treviño et al. 2019; Miyabayashi et al. 2000; Morales‐Espinosa et al. 2009; Zahedi et al. 2017). The main reasons are that the oral bacterial reservoir allows reinfection of gastric tissue, and the antibiotics used as therapy do not eliminate bacteria in the mouth (Adler 2014; Miyabayashi et al. 2000; Zarić et al. 2009; Zou and Li 2011). Some researchers have shown that eradicating oral *H. pylori* with periodontal treatment significantly improves the eradication of stomach infection by systemic treatment (Ozturk 2021; Ren et al. 2023). Also, using mouthwash or antiseptics seems to be a good way to control colonization in the mouth (Kashyap et al. 2020; Song and Li 2013; Wang et al. 2014). The antimicrobial and anti‐biofilm effect against *H. pylori* and the low or absent cytotoxicity of the broad‐spectrum antiseptic, neutral electrolyzed water (NEW) have recently been studied and demonstrated in vitro (Lucio‐Sauceda et al. 2019). According to previous results, the therapeutic use of NEW as a mouthwash in *H. pylori‐*infected patients, along with periodontal treatment and concomitant therapy, seems promising in improving the eradication of gastric and oral infection and recurrence. Herein, we present a clinical trial that evaluated the efficacy of this integral therapy, compared with standard concomitant therapy alone.

## 2 Materials and Methods

### 2.1 Study Design and Population

A prospective, randomized, four‐arm, parallel‐group, open‐label clinical trial was conducted from May 2021 to May 2022. This study compared the efficacy of an integral therapy to eradicate oral and gastric *H. pylori* infection and avoid refractory infections using NEW as mouthwash, periodontal treatment, and concomitant systemic therapy versus standard concomitant systemic therapy alone in double‐positive and symptomatic patients. Patients were recruited from the epidemiology division of the Hospital La Carlota, located in Montemorelos, México. A general invitation was made to patients with gastric symptoms from daily clinical practice. Patients, men, and women 18 years or older who volunteered to participate were evaluated by anamnesis and survey for general data and the presence or absence of clinical symptoms (upper abdominal pain, heartburn with or without reflux, nausea or vomiting, loss of appetite, or early fullness) associated with functional dyspepsia (Rodríguez‐García and Carmona‐Sánchez 2016). Periodontal examination and classification were performed according to the tables by Tonetti, Greenwell, and Kornman (2018). Dental plaque samples were collected to verify *H. pylori* infection in the oral cavity. A stool sample (feces) was required from each patient to verify gastrointestinal tract infection. Both samples were analyzed by qPCR (real‐time or quantitative polymerase chain reaction). Only patients who fulfilled both criteria (symptoms and double‐positive for oral/gastric infection) were included. Patients who received any antibiotic therapy 6 months before the study or were under antitumor/antiviral therapy at the time of the study were excluded. Individuals with autoimmune diseases, immunosuppressed (cancer or presence of tumors), a history or current diagnosis of peptic ulcer, alcoholism, the presence of blood in vomit or feces (digestive bleeding), dysphagia, periodontitis stage III or more, and penicillin allergy or sensitivity to any component of the concomitant therapy were excluded. Patients who did not comply with the follow‐up and/or treatment were eliminated.

### 2.2 Treatment Groups: Integral Therapy and Standard Concomitant Therapy

**Figure 1:**
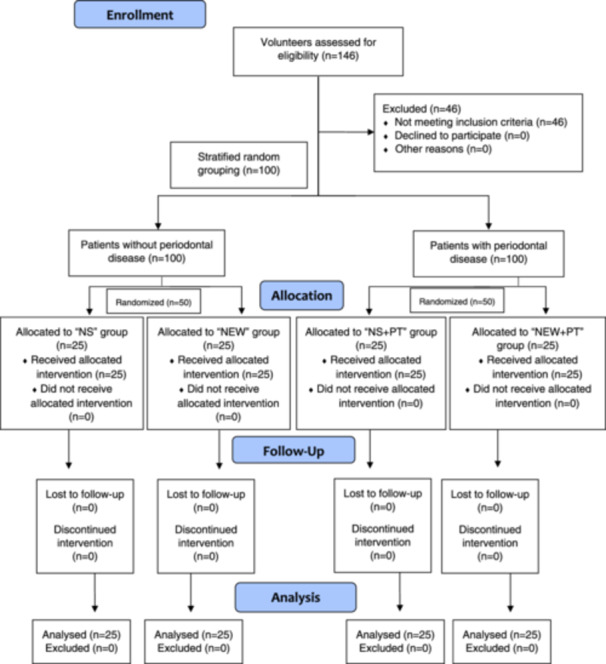
Clinical trial design algorithm. NEW, neutral electrolyzed water; NEW + PT, neutral electrolyzed water + periodontal treatment; NS, normal saline; NS + PT, normal saline + periodontal treatment.

One hundred and forty‐six patients were interviewed for gastric symptoms, examined for periodontal disease, and tested for *H. pylori* double‐positive infection (oral/gastric). Forty‐six patients were excluded since 27 were positive only in the gastric sample and the rest in the oral sample. Stratified random sampling was performed, and 100 patients were equally allocated into two groups: patients without periodontal disease and patients with periodontal disease (stage I/II). Then, each group was randomly subdivided into two additional groups, with four final groups of 25 patients each (Figure 1). Volunteers were randomized according to the order in which they accepted participation; odd numbers were assigned to the control groups (normal saline [NS] as mouthwash) and pair numbers to the treatment groups (NEW as mouthwash). Patients without periodontal disease had no periodontal treatment and received NS (placebo) [NS group] or NEW [NEW group] to perform mouthwash. Patients with periodontal disease had periodontal treatment (PT) and received placebo mouthwash (NS) [NS‐PT group] or NEW mouthwash [NEW‐PT group]. All patients had standard non‐bismuth quadruple systemic therapy (concomitant therapy) every 12 h for 10 days with proton pump inhibitor (omeprazole 40 mg), clarithromycin (500 mg), amoxicillin (1000 mg), and metronidazole (500 mg) (Bosques‐Padilla et al. 2018).

### 2.3 Periodontal Therapy

Periodontal therapy included scaling and root planning. Treatment was performed in all patients with periodontal disease just before the start of systemic treatment. It included manual instrumentation of the teeth to remove dental plaque and calculus from the affected enamel and cementum, ultrasonic scaling therapy, flossing, irrigation, and oral hygiene instructions. Standard irrigation with 0.12% chlorhexidine mouthwash for 45 s was substituted with NEW. Oral hygiene information included the use of the designated mouthwash substance per group.

### 2.4 Mouthwash Substances: NEW and NS

The broad‐spectrum antiseptic, NEW (pH 6.5−7.5; REDOX potential 750−950 mV; 0.0015% of active species of chlorine and oxygen) was provided by Esteripharma S.A. de C.V (commercial product name, OXORAL, COFEPRIS registration no. 2072C2009 SSA). NS solution (aqueous sodium chlorine 0.9% w/v) was used as a placebo. Mouthwash was indicated for 30 s, three times a day, with 15 mL of the corresponding liquid each time for 10 days. All participants were informed of the importance of following the protocol and the correct way to perform the mouthwash.

### 2.5 Outcome Measures and Follow‐Up

Three primary endpoints were established. The first was eliminating gastric *H. pylori* infection (gastric‐negative patients, with or without oral infection) 4 weeks after treatment. The second was the absence of refractory *H. pylori* gastric infection (gastric‐negative patients, with or without oral infection) 16 weeks after therapy. For this purpose, only gastric‐negative patients (with or without oral infection) from the first primary endpoint were considered and evaluated. Two new groups were formed: patients from the NS and NS‐PT groups [NS + NS‐PT group] or patients from the NEW and NEW‐PT groups [NEW + NEW‐PT group]; patients from the new groups were tested for infection 12 weeks later. The third primary endpoint was the success of the integral therapy, where an integral therapy was considered successful if no gastric infection or associated symptoms were detected 16 weeks after treatment. Secondary endpoints were improvement or remission of symptoms per treatment group, 4 and 16 weeks posttreatment. Symptom improvement was considered when at least two of four symptoms were eradicated after treatment. Symptom remission was considered when all symptoms were eradicated after treatment (asymptomatic patients).

Before starting each integral therapy, all patients were confirmed as symptomatic (the presence of at least three gastric symptoms) and double‐positive *H. pylori*‐infected (oral and gastric infection). As a follow‐up, subgingival dental plaque and stool samples were obtained 4 and 16 weeks after the end of each therapy to detect and quantify *H. pylori* by real‐time PCR. Symptom evolution was also monitored with a survey 4 and 16 weeks after each therapy. Only gastric‐negative or double‐negative patients were considered for the 16‐week follow‐up of recurrence and change in symptom status. *H. pylori* infection recurrence and symptom change were evaluated by grouping and comparing patients that did not have NEW mouthwash (NS and NS‐PT groups) versus patients with NEW mouthwash (NEW and NEW‐PT groups). Patients with positive gastric infection (*H. pylori* detection in gastric samples with or without oral infection) after the first follow‐up were provided with NEW mouthwash and advised to consult their physician for alternative systemic therapies. Further follow‐up of these patients was not performed.

### 2.6 Sample Collection

#### 2.6.1 Oral Sample

Dental plaque was collected with a sterile bamboo toothpick by scraping downward against the buccal (supragingival) and subgingival surfaces of the upper and lower first molars. The sample was suspended in 1.5 mL trypticasein soy broth (Becton Dickinson, Franklin Lakes, NJ) supplemented with 30% glycerol to maintain microbial viability and then refrigerated at −70°C until use.

#### 2.6.2 Gastrointestinal Sample

A stool sample was obtained from each patient before treatment and 1 and 4 months after concluding the treatment. The patients collected the samples according to the tube stool sample collection system (Cat#45660, Norgen Biotek CO, Thorold, ON, Canada). The samples were transported to the clinical analysis laboratory of Montemorelos University and stored at 4°C until total DNA extraction.

### 2.7 DNA Extraction

#### 2.7.1 Dental Plaque Sample

The dental plaque samples were centrifuged at 12,000×*g* for 2 min, washed with PBS pH 7.4, and suspended in 100 μL of Tris‐EDTA buffer (10 mM Tris and 1 mM EDTA, pH 7.4). For enzymatic cell lysis, 10 μL of lysozyme (10 mg/mL) and 10 μL of Proteinase K (10 mg/mL) were added and incubated at 56°C for 30 min. Total DNA was extracted using a High Pure PCR kit (Roche Diagnostics, GmbH, Mannheim, Germany) according to the manufacturer's recommendations. The DNA concentration was measured at 260 nm in a spectrophotometer (NanoDrop 8000 UV‐Vis; Thermo Scientific, Wilmington, DE). DNA samples were stored at −20°C until use.

#### 2.7.2 Stool Sample

The Stool DNA Isolation Kit (Cat#27600, Norgen Biotek CO; Thorold, ON, Canada) was used according to the manufacturer's recommendations to extract total DNA from a 200 mg feces sample.

### 2.8 Detection and Quantification of *H. pylori* by Real‐Time PCR

A calibration curve for the *H. pylori* 16S rRNA gene was constructed from a pGEMT‐easy vector cloned with the gene fragment. The number of plasmid copies per microliter (cg/μL) was calculated. Ten serial dilutions were made in base 10 and analyzed in triplicate by real‐time PCR to construct calibration curves. Positive and negative results of the 16S rRNA gene were obtained for the oral cavity and stool samples by interpolating *C*_t_ values on the standard curve. The presence of *H. pylori* was assessed by qPCR using oligonucleotides and probes previously designed for the 16S rRNA gene (Urrutia‐Baca et al. 2018). The qPCR reactions were performed in 96‐well plates containing 12.5 μL of 2× qPCR master mix (Thermo Scientific, Carlsbad, CA, USA), 0.3 μM oligonucleotides, 0.2 μM probe, and 100 ng of DNA from the sample and water to a final volume of 25 µL. A total of 100 ng of DNA from *H. pylori* strains ATCC700824 or ATCC43504 and nuclease‐free water were added as positive and negative controls. The qPCR assay was carried out in a LightCycler 480II thermal cycler (Roche, Mannheim, Germany) programmed with a single color format (6‐FAM, filter combination 465‐510): one denaturation cycle (95°C, 10 min), 35 amplification cycles (95°C, 10 s, 4°C/s ramp, 55°C, 15 s, 2°C/s ramp, 72°C, 15 s, 4°C/s quantitation analysis ramp rate), one melting cycle (95°C, 5 s, ramp rate of 4°C/s, 65°C, 1 min, ramp of 2.2°C/s, 97°C with continuous acquisition of 5°C), and a cooling cycle (40°C, 10 s, 1.5°C/s ramp).

### 2.9 Ethical Considerations

The study was approved by the institutional ethics committee and the Health Sciences Research and Development Center (CIDICS) of the Autonomous University of Nuevo Leon (UANL) [January 2020; SPSI‐010613; No. 00208] and was conducted in accordance with the international ethical standards established in the Declaration of Helsinki. The purpose and procedures of the study were explained to all participants. The results were confidential and were only used for scientific research purposes. Written informed consent was obtained from each participant before the study. The clinical protocol was approved just before the COVID‐19 pandemic in Mexico; therefore, patient recruitment was postponed until safety conditions were appropriate to continue 16 months later.

### 2.10 Blinding

The researchers who performed the statistical analyses were blinded.

### 2.11 Statistical Methods

IBM SPSS Statistics for Windows, version 29.0.1.0 (171), was used for data processing. Age values are expressed as mean ± standard deviation (data with a normal distribution according to Kolmogorov−Smirnov) and were compared between groups using one‐way ANOVA (*α* = 0.05). The rest of the data are expressed as the number and percentage of incidences. Categorical variables were compared using Fisher's exact test or the Pearson *χ* ^2^ test. The Wilcoxon signed‐rank test was used as a nonparametric statistical analysis to evaluate significance within the same group before and after treatment. Relative risk (RR) and number needed to treat (NNT) were calculated using the MedCalc Software Ltd. RR calculator, Version 22.007 (MedCalc Software, 2023). A *p* < 0.05 value was statistically significant and was calculated as a two‑tailed test for all statistical analyses.

## 3 Results

### 3.1 General Data, Periodontal Disease, and Symptoms

One hundred patients met the criteria and were selected and included in the trial. Data regarding gender, age, gastric symptoms, and presence/absence of periodontal disease were collected and analyzed. Mean age of 43.34 ± 14.07 years was recorded for all participants, with 66% (66/100) of the individuals being women and 34% (34/100) men. Regarding clinical symptoms, 94% (94/100) of all participants experienced upper abdominal pain, while 96% (96/100) had heartburn with or without reflux, vomiting or nausea, and early satiety when eating. Periodontal disease was present in 50% of included patients; 64% (32/50) were diagnosed as stage I, while the rest (36% [18/50]) were stage II. No significant differences were found regarding age (*p* = 0.115), gender (*p* = 0.828), or gastric symptoms (*p* > 0.87) among the groups, nor in the periodontal disease stage between the groups, NEW‐PT and NS‐PT (*p* = 0.247) (Table 1).

**Table 1:** General characteristics of enrolled individuals: Age, gender, periodontal disease, and gastric symptoms.

Group (*N*)	Age, mean ± SD	Gender, *N* (%)		Periodontal disease, *N* (%)		Gastric symptoms, *N* (%)			
		Male	Female	Stage I	Stage II	Upper gastric pain	Heartburn	Nausea/vomiting	Early satiety
NS (*N* = 25)	40.20 ± 12.05	32 (8)	68 (17)	0	0	92 (23)	96 (24)	96 (24)	96 (24)
NEW (*N* = 25)	44.40 ± 12.32	36 (9)	64 (16)	0	0	96 (24)	96 (24)	96 (24)	96 (24)
NS‐PT (*N* = 25)	48.48 ± 17.50	40 (10)	60 (15)	56 (14)	44 (11)	92 (23)	96 (24)	96 (24)	96 (24)
NEW‐PT (*N* = 25)	40.28 ± 12.83	28 (7)	72 (18)	72 (18)	28 (7)	96 (24)	96 (24)	96 (24)	96 (24)
Mean values	43.34 ± 14.07	34 (34/100)	66 (66/100)	64 (32/50)	36 (18/50)	94 (94/100)	96 (96/100)	96 (96/100)	96 (96/100)
a *p* value	0.115^b^	0.828		0.2473		0.871	1	1	1

Abbreviations: NEW, neutral electrolyzed water; NEW‐PT, mouthwash with NEW and periodontal treatment; NS, mouthwash with normal saline; NS‐PT, mouthwash with normal saline and periodontal treatment.

^a^ Statistical significance according to Pearson's *χ* ^2^.

^b^ One‐way ANOVA; *α* = 0.05.

### 3.2 Eradication of *H. pylori* Infection and Improvement of Symptoms 4 Weeks After Treatment

The success in eradicating *H. pylori* infection, generally and per group 4 weeks after treatment completion, is indicated in Table 2. A significant difference (*p* < 0.001) was observed in the success of eradicating gastric infection between the NS and NEW groups and the NS‐PT and NEW‐PT groups. Concomitant treatment plus NEW mouthwash was better than concomitant treatment alone for individuals without or with periodontal disease, with 20% (5/25) versus 84% (21/25) and 56% (14/25) versus 96% (24/25) of gastric‐negative patients, respectively. On the other hand, a significant difference was also found (*p* = 0.009) when the comparison was made between the NS and NS‐PT groups, both without NEW mouthwash. Periodontal treatment was more effective (20% vs. 56%) than concomitant treatment alone (NS group) in increasing the eradication rate of gastric *H. pylori*. A significant difference (*p* < 0.001; data not shown) was found in the efficacy analysis for all treatments, either in the elimination of gastric infection or the elimination of gastric and oral infection (not infected patients), with 64% and 58% of negative cases, respectively.

**Table 2:** Detection of oral and/or gastric *H. pylori* infection 4 weeks after treatment.

Group	*H. pylori* detection by qPCR, *N* (%)				Negative gastric infection	*p* values^a^ (negative gastric infection)			Not infected patients	*p* values^a^ (not infected patients)		
	Oral (+) gastric (+)	Oral (+) gastric (−)	Oral (−) gastric (+)	Oral (−) gastric (−)								
NS (*N* = 25)	19 (76%)	2 (8%)	1 (4%)	3 (12%)	5 (20%)				3 (12%)			
NEW (*N* = 25)	3 (12%)	1 (4%)	1 (4%)	20 (80%)	21 (84%)	< 0.001***			20 (80%)	< 0.001***		
NS‐PT (*N* = 25)	6 (24%)	0	5 (20%)	14 (56%)	14 (56%)	0.009**	0.031*		14 (56%)	0.001***	0.069	
NEW‐PT (*N* = 25)	1 (4%)	3 (12%)	0	21 (84%)	24 (96%)	< 0.001***	0.349^b^	< 0.001***	21 (84%)	< 0.001***	1^b^	0.031*
Total	29 (29%)	6 (6%)	7 (7%)	58 (58%)	64 (64%)				58 (58%)			

Abbreviations: NEW, neutral electrolyzed water; NEW‐PT, mouthwash with NEW and periodontal treatment; NS, mouthwash with normal saline; NS‐PT, mouthwash with normal saline and periodontal treatment.

^a^ Statistical significance according to Pearson's *χ* ^2^.

^b^ Fisher exact test.

* *p* = 0.05

** *p* = 0.01

*** *p* ≤ 0.001.

With respect to symptom improvement, the efficacy of each treatment was NEW‐PT > NEW > NS‐PT >>> NS. Generally, symptoms prevailed in only 4%–8% of individuals from the group NEW‐PT, while a prevalence of 8%–16% was observed in individuals from group NEW. In groups NS‐PT and NS, symptoms persisted in 24%–44% and 48%–88% of patients, respectively (Table 3). Concomitant therapy and the use of NEW as mouthwash, with or without periodontal therapy, eliminated at least two symptoms in 92% (NEW‐PT) or 88% (NEW) of patients versus 68% in NS‐PT or 24% in NS groups. No significant improvement was observed after treatment in the NS group, except for nausea/vomiting (*p* = 0.006), unlike the rest of the groups (*p* < 0.001) (Table 3). Among groups, both NEW and NEW‐PT integral treatments were equally effective in reducing or eradicating symptoms associated with infection since no significant differences were observed between them (Table 3).

**Table 3:** Presence of symptoms associated with *H. pylori* infection before and 4 weeks after treatment.

Group (*N*)	Pain in the upper chest						Heartburn						Nausea/vomiting					
	Before treatment	4 W after treatment	*p* value^a^	*p* value^b^			Before treatment	4 W after treatment	*p* value^a^	*p* value^b^			Before treatment	4 W after treatment	*p* value^a^	*p* value^b^		
NS (*N* = 25)	23 (92%)	19 (76%)	0.125				24 (96%)	19 (76%)	1				24 (96%)	12 (48%)	0.006**			
NEW (*N* = 25)	24 (96%)	2 (8%)	< 0.001***	< 0.001***			24 (96%)	4 (16%)	< 0.001***	< 0.001***			24 (96%)	4 (16%)	< 0.001***	0.015*		
NS‐PT (*N* = 25)	23 (92%)	8 (32%)	< 0.001***	0.002**	0.034*		24 (96%)	10 (40%)	< 0.001***	0.01**	0.059		24 (96%)	6 (24%)	< 0.001***	0.077	0.48	
NEW‐PT (*N* = 25)	24 (96%)	2 (8%)	< 0.001***	< 0.001***	1^c^	0.034*	24 (96%)	2 (8%)	< 0.001***	< 0.001***	0.384	0.008**	24 (96%)	2 (8%)	< 0.001***	0.002*	0.667^c^	0.247^c^
Total	94 (94%)	31 (31%)					96 (96%)	35 (35%)					96 (96%)	24 (24%)				

Early satiety					
Before treatment	4 W after treatment	*p* value^a^	*p* value^b^		
24 (96%)	22 (88%)	0.5			
24 (96%)	3 (12%)	< 0.001***	< 0.001***		
24 (96%)	11 (44%)	< 0.001***	< 0.001***	0.012*	
24 (96%)	1 (4%)	< 0.001***	< 0.001***	0.609^c^	< 0.001***
96 (96%)	37 (37%)				

Abbreviations: NEW, neutral electrolyzed water; NEW‐PT, mouthwash with NEW and periodontal treatment; NS, mouthwash with normal saline; NS‐PT, mouthwash with normal saline and periodontal treatment; 4 W, 4 weeks.

^a^ Statistical significance according to Wilcoxon signed‐rank test.

^b^ Statistical significance according to Pearson's *χ* ^2^.

^c^ Fisher exact test.

* *p* = 0.05

** *p* = 0.01

*** *p* ≤ 0.001.

### 3.3 Eradication of *H. pylori* Infection and Associated Symptoms 16 Weeks After Treatment: Recurrence and Integral Therapy Success

After treatment completion, all patients with negative gastric infection were regrouped into two: patients treated [NEW‐PT and NEW groups] or untreated [NS‐PT and NS groups] with NEW mouthwash. The group of patients treated with NEW mouthwash included 45 individuals: 24 from the NEW‐PT group and 21 from the NEW group. The group of untreated patients with NEW mouthwash included 19 individuals: 14 from the NS‐PT group and five from the NS group. The follow‐up data were analyzed 16 weeks after treatment completion, and a significant difference in recurrence was observed (*p* = 0.001). Recurrence was higher in patients who did not perform mouthwash with NEW; 47.4% (9/19) of patients in the NS + NS‐PT group presented infection, while only 8.9% (4/45) of patients that received NEW mouthwash showed gastric infection (Table 4). More notably, 86.7% (39/45) of individuals treated with NEW mouthwash were not infected at all (bacteria not detected in stomach or mouth) 16 weeks after treatment, compared with only 26.3% (5/19) of not infected individuals in the other group (*p* ≤ 0.001; data not shown). Additionally, the RR analysis (Table 4) revealed a protective effect of practicing oral hygiene with NEW mouthwash, with or without periodontal treatment, to avoid gastric *H. pylori* reinfection in 81.2% (RR = 0.1877; 95% CI: 0.0658−0.5355; *p* = 0.0018), confirming the importance of eradicating and controlling bacteria in the oral cavity. Symptom analysis also revealed a significant difference between treatments for asymptomatic individuals, with a *p* ≤ 0.001 for upper gastric pain, heartburn, and early satiety and a *p* = 0.003 for nausea/vomiting (Table S1).

**Table 4:** Incidences and relative risk of gastric recurrences of *H. pylori* infection 16 weeks after treatment.

Groups [*N*]	Positive, *N* (%) gastric infection	Negative, *N* (%) no gastric infection	*p* value^a^	Relative risk (95% CI)	*p* value^a^
NS + NS‐PT	9 (47.4%)	10 (52.6%)	0.001***	0.1877 (0.0658−0.5355)	0.0018**
[*N* = 19]					
NEW + NEW‐PT	4 (8.9%)	41 (91.1%)			
[*N* = 45]					
Total	13 (20.3%)	51 (79.7%)			

Abbreviations: NEW, neutral electrolyzed water; NEW‐PT, mouthwash with NEW and periodontal treatment; NS, mouthwash with normal saline; NS‐PT, mouthwash with normal saline and periodontal treatment.

^a^ Statistical significance according to Pearson's *χ* ^2^ between positive and negative individuals.

** *p* ≤ 0.001

*** *p* = 0.01.

At the end of the follow‐up, per‐protocol (PP) and intention‐to‐treat (ITT) analyses were performed regarding therapy success (Table 5). Therapy was successful when no gastric infection nor associated symptoms were detected 16 weeks after treatment. Only 47.4% (PP) or 18% (ITT) of patients treated without NEW as mouthwash (concomitant therapy + oral rinses with neutral saline, with or without periodontal therapy) met both conditions. In contrast, 88.9% (PP) or 80% (ITT) of patients who had systemic medication and cared for their oral health with NEW rinses, with or without periodontal therapy, were asymptomatic and negative for gastric infection at the end of the follow‐up. The success of these integral therapies was ≥80%. Even more, a RR = 0.2439 (*p* < 0.001; 0.1380−0.4310, 95% CI) based on ITT data was calculated, indicating a significant protective effect of 76.6% for the patients that took care of oral hygiene together with systemic treatment, to avoid infection recurrence and associated symptoms after completion of the integral therapy, compared with individuals not treated with NEW mouthwash and who only received systemic therapy and mouthwash with NS with or without periodontal treatment. The NNT was 1.613 (*p* < 0.001; 1.292−2.145, 95% CI) (Table 5). These results show the impact on oral health achieved with an effective antiseptic mouthwash and the performance of periodontal treatment in the case of periodontal disease, together with systemic treatment as integral therapy to eradicate gastric *H. pylori* infection, its associated symptoms, and prevent recurrence and complications.

**Table 5:** Integral therapy success after 16 weeks of follow‐up.

Group [*N*_PP_; *N*_ITT_]	Asymptomatic, *N* (%)	Negative for gastric infection, *N* (%)	Successfully treated (PP), *N* (%)	Successfully treated (ITT), *N* (%)	*p* value^a^	Relative risk^b^ (95% CI)	NNT (benefit)^b^ (95% CI)
NS + NS‐PT [*N*_PP_ = 19; *N*_ITT_ = 50]	9 (47.4)	10 (52.6)	9 (47.4)	9 (18)	0.001***	0.2439 (0.1380−0.4310)	1.613 (1.292−2.145)
NEW + NEW‐PT [*N*_PP_ = 45; *N*_ITT_ = 50]	40 (88.9)	41 (91.1)	40 (88.9)	40 (80)			

Abbreviations: ITT, intention‐to‐treat analysis; *N*_ITT_, number of individuals considered for intention to treat analysis; *N*_PP_, number of individuals considered for per‐protocol analysis; NEW, neutral electrolyzed water; NEW‐PT, mouthwash with NEW and periodontal treatment; NS, mouthwash with normal saline; NS‐PT, mouthwash with normal saline and periodontal treatment; PP, per‐protocol analysis.

^a^ Statistical significance according to Pearson's *χ* ^2^ between positive and negative individuals.

^b^ Relative risk and number needed to treat (NNT) were calculated and expressed as an intention‐to‐treat (ITT) analysis.

*** *p* ≤ 0.001.

## 4 Discussion

The gastric eradication rate in our study, 4 weeks after treatment, was below the accepted efficacy rate (≥80%) and also below the 80%–95% efficacy reported in international clinical trials or worldwide meta‐analyses for concomitant therapy alone (Gisbert and Calvet 2012; Li et al. 2015; Liou et al. 2016; Mestrovic et al. 2020). However, it is important to point out that the individuals evaluated in such studies were not Mexican or Latin American populations, and the existence of major differences in resistance is well documented by geographic regions affecting eradication rates (Escobedo‐Belloc and Bosques‐Padilla 2019; Malfertheiner et al. 2023).

A meta‐analysis with 1463 individuals from Latin America revealed success rates of 78.7% (per protocol) and 73.6% (intention to treat) after 5 days of concomitant treatment. More specifically, the efficacy rates for the Mexican population (*N* = 420) were 68.1%–72.5% (Greenberg et al. 2011), which are closer to the efficacies observed in this trial. Unfortunately, no additional trials regarding concomitant therapy efficacy in the Mexican population have been developed; instead, second‐line quinolone‐based therapies (levofloxacin, ofloxacin) were explored, showing a low efficacy of 62%–63% when used for 7 days. An efficacy rate of 92.3% was reported after 14 days of ofloxacin‐based therapy (Bosques‐Padilla et al. 2004; Ladrón‐de‐Guevara et al. 2019). The remarkable low efficacy of first‐ and second‐line therapies in Mexico is due to the high antibiotic resistance that has increased over time. Clarithromycin‐based triple therapy is no longer recommended, nor should azithromycin‐based therapy, since local clarithromycin resistance is above 15% (Alarcón‐Millán et al. 2016; Bosques‐Padilla et al. 2018; Escobedo‐Belloc and Bosques‐Padilla 2019). Clarithromycin, metronidazole, and amoxicillin resistance reported in 2014 for *H. pylori* isolates from the Mexican population were 13%, 60%, and 4%, respectively (Camargo, García, and Riquelme 2014). A recent study showed increased antibiotic resistance in *H. pylori* strains (*N* = 111) isolated from Mexican individuals (diagnosed with non‐atrophic gastritis, intestinal metaplasia, gastric cancer, or duodenal ulcer) over 20 years. This study found that 85% of the strains were resistant to at least one antibiotic. According to this analysis, from 2011 to 2017, clarithromycin resistance increased from 11.5% to 32.2%, levofloxacin from 30.8% to 58.1%, amoxicillin from 0% to 6.5%; metronidazole decreased from 73% to 51.6% in analyzed samples. Double or triple resistance was also detected in strains from 2017 (*N* = 31), with resistance rates of 32% for metronidazole + levofloxacin, 25.8% for clarithromycin + metronidazole, and 4.5% for metronidazole + levofloxacin + clarithromycin (Camorlinga‐Ponce et al. 2020). Despite the small size of the analyzed samples in 2017, it was useful to prove the increased antibiotic resistance in Mexico, together with Camargo's analysis, and to explain in part the low efficacy rates of eliminating *H. pylori* infection with standard first‐ and second‐line empirical treatments. It is strongly suggested that for countries like Mexico, where antibiotic resistance is high, antibiotic susceptibility testing must be performed before an empiric therapeutic regimen. If there is no access to such tests, management based on the history of medications used in the past could be a suitable alternative (Alarcón‐Millán et al. 2016; Bosques‐Padilla et al. 2018; Escobedo‐Belloc and Bosques‐Padilla 2019; Fallone, Moss, and Malfertheiner 2019; Malfertheiner et al. 2023).

With the high antibiotic resistance in the Mexican population, another variable to consider for the lower eradication rates in our study is that all patients were symptomatic and doubly colonized (oral and gastric). Similar research in these patients shows that it is more complicated to eradicate gastric infection than in patients without oral infection (Gao et al. 2011; Miyabayashi et al. 2000; Song and Li 2013; Zarić et al. 2009). Many authors have demonstrated that poor oral hygiene and a lack of bacterial eradication in the oral cavity may be the main reason for systemic therapy failure, recurrence, and resistant *H. pylori* strains (Abadi et al. 2014; Adler 2014; Al Sayed et al. 2014; Anand, Kamath, and Anil 2014; Bouziane et al. 2012; Gao et al. 2011; Jia et al. 2009; Miyabayashi et al. 2000; Song and Li 2013; Wang et al. 2014; Yee 2016; Zarić et al. 2009; Zhang et al. 2022; Zou and Li 2011).

The oral cavity is the gate to the gastric tract, and the fact that *H. pylori* are present in the oral cavity, in the oral mucosa, tongue, dental plaque, saliva, supra‐ and subgingival plaque, has been extensively studied and demonstrated. Thus, the oral cavity has been identified as an extra‐gastric reservoir of *H. pylori* that actively participates in bacteria transmission from the mouth to the stomach, complicating the elimination of gastric infection (Adler 2014; Al Sayed et al. 2014; Anand, Kamath, and Anil 2014; Bouziane et al. 2012; Gao et al. 2011; Jia et al. 2009; Zarić et al. 2009; Zou and Li 2011). Nonetheless, controversy about the true existence of bacteria in the oral cavity, as part of the complex oral microenvironment and with the potential to cause gastric infection and disease, persists (Mao et al. 2021; Navabi, Mirzazadeh, and Aramon 2011). Multiple research studies and analyses have offered evidence about its plasticity (spiral to coccoid morphology and vice versa) and ability to prevail in the oral cavity, especially in dental plaque as part of the complex oral subenvironment, and its capacity to induce infection and disease once it has reached gastric tissue (Gao et al. 2011; Kadkhodaei, Siavoshi, and Akbari Noghabi 2020; Mao et al. 2021; Zhang et al. 2022). In this sense, the Maastricht VI Florence consensus report recognizes that the oral cavity may contribute to the gastric microbiota composition through swallowing acts that transfer oral bacteria to the stomach (Malfertheiner et al. 2022).

Nevertheless, no recommendations about periodontal treatment or antiseptic mouthwash as part of an integral treatment (oral hygiene and systemic therapy) have been published. To the best of our knowledge, no international guideline related to gastric *H. pylori* eradication regimens includes oral health and hygiene (periodontal treatment, use of mouthwash, and eradication of periodontal disease) as part of the integral therapies, despite multiple related reviews, recent or carried out for more than a decade (Al Sayed et al. 2014; Alkhaldi et al. 2022; Anand, Kamath, and Anil 2014; Bouziane et al. 2012; Ozturk 2021; Ren et al. 2016; Zou and Li 2011). The idea that it is more necessary to develop new technologies and/or integral therapies, including oral hygiene, than new antibiotics has been postulated. According to their results, many authors strongly believe that cleaning the oral cavity from *H. pylori* and promoting regular oral hygiene may allow clinicians to prescribe current systemic therapies with better efficacy and eventually reduce the number of prescribed drugs (Abadi et al. 2014; Al Sayed et al. 2014; Anand, Kamath, and Anil 2014; Bouziane et al. 2012; Butt et al. 2001; Gao et al. 2011; Jia et al. 2009; Song and Li 2013; Wang et al. 2014; Yee 2016; Zarić et al. 2009; Zou and Li 2011). We agree with these ideas. Our study concludes that the eradication rate of *H. pylori* with concomitant systemic therapy alone significantly increases (more than fourfold) if an effective and safe antiseptic such as NEW is used as a mouthwash (*p* < 0.001). The increased efficacy is even better (96%) if periodontal treatment is performed before starting medication (*p* < 0.001). Our results agree with previous findings in similar clinical trials with double‐colonized patients, where the eradication rate of gastric *H. pylori* with systemic treatment increased from 61.5% to 82.3% (Wang et al. 2014) when a mouthwash was indicated together with systemic therapy, or from 78.4% to 94.7% when, in addition to mouthwash, periodontal treatment was performed (Song and Li 2013). Other authors evaluated the effect of periodontal treatment combined with systemic therapy and found significantly better gastric eradication rates than systemic therapy alone (Gao et al. 2011; Zarić et al. 2009). The corresponding result in our trial (group NS vs. NS‐PT; *p* = 0.009) agrees with this result, but it was not as good as the one obtained when mouthwash was added (NEW and NEW‐PT groups vs. NS and NS‐PT groups). Even more, double elimination of the bacteria in the mouth and stomach in the NEW and NEW‐PT groups also reached acceptable rates of 80% (20/25) and 84% (21/24), respectively (Table 2). The benefit of including oral hygiene regimens, such as periodontal treatment, mouthwash, and systemic therapy, is undeniable. On the other hand, as was expected, together with the elimination of gastric infection, an improvement in associated gastric symptoms was observed in 88%–92% of patients from groups treated with NEW mouthwash compared to 24%–68% in groups not treated with NEW mouthwash.

Our trial included a 16‐week follow‐up of patients who achieved gastric infection eradication after the first follow‐up to investigate the efficacy of the different treatments for preventing recurrence and achieving the cure of the disease. The literature describes a worldwide recurrence rate of 4%–9% and up to 13% for developing countries. Rates of up to 11.5% have been described in Latin America and in Mexico of up to 18.8% (Bosques‐Padilla et al. 2018; Malfertheiner et al. 2023; Niv 2008; Zhao et al. 2021). Our data revealed significant differences (*p* = 0.001) in favor of patients who used NEW mouthwash with or without periodontal therapy, with 8.9% (4/45) of gastric reinfection in comparison with the other groups, where recurrences were as high as 47.4% (9/19). Significant differences were found between all groups regarding symptom eradication. Both results, infection and symptom eradication, indicate a difference in the success of therapies, with those groups treated with NEW mouthwash with or without periodontal treatment being significantly better (*p* = 0.001) (Table 5).

Although our percentages were above the numbers reported for Latin America and Mexico, they agree with other studies that included similar types and numbers of patients. Jia et al. (2009), who included symptomatic patients recently cured of gastric infection with systemic therapy, demonstrated that the practice of oral hygiene (periodontal therapy and mouthwash) in individuals successfully cured infection and prevented gastric reinfection 6 months later (19.6% of positive cases; *N* = 11/56) compared to individuals without oral hygiene practices (84.3% of positive cases; *N* = 43/51) (Miyabayashi et al. 2000). Another study showed that symptomatic and double‐positive patients treated with triple therapy and periodontal treatment had fewer recurrences of gastric infection than those treated only with systemic therapy 1 year after treatment (Gao et al. 2011). These facts offer strong evidence to conclude that oral hygiene is critical in eradicating gastric infection and/or preventing infection or recurrence. This statement matches the results of a long‐term study (Miyabayashi et al. 2000), where 69.5% (16/23) of gastric and orally colonized patients treated with systemic therapy were negative at 2 years of follow‐up compared with 95.8% (23/24) of gastric‐negative patients who were not orally colonized from the beginning.

Additionally, a recent epidemiological study with information from almost 135,000 Taiwanese individuals collected over 13 years demonstrated that those with periodontal disease had a higher risk of developing gastric *H. pylori* infection than those without periodontitis (Li et al. 2021). Furthermore, recent research on Mexican adults and children regarding periodontal disease severity revealed an associated risk of carrying virulent *H. pylori* strains (Flores‐Treviño et al. 2019; Mendoza‐Cantú et al. 2017). Again, oral hygiene seems to be the clue to improving the success of systemic therapies against gastric *H. pylori* infection and preventing complications.

Most of the analyzed studies that included antiseptic irrigation of the oral cavity as part of the periodontal therapy or as mouthwash recommended chlorhexidine gluconate 0.12% (1200 ppm of active ingredient). For decades, this chemical agent has been considered a good short‐term substance to prevent oral infections after oral procedures and eliminate oral diseases, such as dental plaque, periodontitis, and gingivitis, due to its antimicrobial properties (Najafi et al. 2012; Ren et al. 2023). Nonetheless, its cytotoxicity (Müller et al. 2014; Torres‐Capetillo et al. 2013) and potential to induce adverse effects, such as mouth irritation, tooth staining (when used for 4 or more weeks), dry mouth, altered taste (unpleasant and/or decreased‐increased taste), discolored or coated tongue, increased calculus formation, desquamation of the oral mucosa, and on very rare occasions, swelling of the face, lips, tongue, or throat due to anaphylactic reactions (Brookes et al. 2020), have been well established. Moreover, some authors point out that there is not enough evidence to affirm that chlorhexidine mouthwash helps reduce gingivitis in moderate or severe levels of gingival inflammation. However, strong evidence points to its efficacy in reducing dental plaque when used as an adjunct to periodontal treatment (James et al. 2017). Other authors have identified that multiple pathogenic bacteria might develop important chlorhexidine resistance (Buxser 2021; Kampf 2016). Another important fact concerning chlorhexidine's antimicrobial effect, instantaneous or residual, is its potential to cause dysbiosis in the oral microenvironment in the short term (5–7 days of use). Recent research shows that chlorhexidine mouthwash alters the oral microbiome, particularly nitrate‐reducing bacteria, leading to lower nitrite availability in healthy individuals (Bescos et al. 2020). Higher glucose and lactate concentrations in saliva and lower nitrate concentrations, nitrite‐reduced buffering capacity, and pH were also reported after 7 days of chlorhexidine mouthwash (Brookes et al. 2021). Such effects were also observed in hospitalized individuals who performed oral rinses twice daily for 5–7 days (Liu et al. 2023). Another study showed that the imbalance in nitrate‐reducing bacteria favors the progression of caries and may produce other complications since these bacteria are part of the normal flora and are considered probiotics that help maintain appropriate systemic nitric oxide levels (Rosier et al. 2020, 2022).

Our study demonstrated the convenience of systemic therapy and antiseptic mouthwash, with or without periodontal treatment, according to the presence or absence of periodontal disease, to increase the success rate of gastric *H. pylori* eradication and improve associated symptoms. Even more, a significant protective effect of 76.6% was calculated for the patients who performed oral hygiene with mouthwash and periodontal treatment versus those with only systemic treatment to avoid infection and disease recurrence 4 months after completing integral therapy. Other works have shown that oral hygiene, in the long term, prevents infections or reinfection when *H. pylori* prevails in the oral cavity (Jia et al. 2009). Considering that recommended therapies last from 7 to 15 days and that in countries with a high index of antibiotic resistance, some patients must be retreated, it is a good recommendation to incorporate a safe and effective oral antiseptic to ensure oral hygiene during systemic therapy without potential adverse effects in the long term. Unlike chlorhexidine gluconate, a xenobiotic substance, NEW is an effective and nontoxic broad‐spectrum antiseptic since its active ingredients, 0.0015% (15 ppm) of active species of chlorine and oxygen (e.g., HOCl), are physiologically synthesized by the human immune system during the innate response against infections and during injuries (Akl 2023). Its efficacy and safety in multiple medical applications, especially in wound healing, owing to its anti‐inflammatory and epithelizing properties and over soft tissue and mucosa to prevent or eliminate infection, have been demonstrated (Cárdenas et al. 2022; Delgado‑Enciso et al. 2021; Gutiérrez‐García et al. 2022; Medina‐Tamayo et al. 2007; Reis et al. 2021; Yan, Daliri, and Oh 2021). Particularly, the efficacy and safety of NEW in periodontal procedures have been demonstrated (Cárdenas et al. 2022; González‐Cantú et al. 2022; Lucio‐Sauceda et al. 2019; Sam and Lu 2009). A study conducted with patients positive for periodontal disease showed improvement in plaque index, gingival index, and probing depth after periodontal therapy with NEW as an irrigating solution and mouthwash (González‐Cantú et al. 2022). Particularly in *H. pylori*, the antimicrobial and anti‐biofilm effects of NEW were demonstrated with gene expression experiments (Lucio‐Sauceda et al. 2019). NEW, as mouthwash and during periodontal treatment, seems to be a good option to promote oral hygiene and a convenient complement to systemic therapies to eradicate gastric *H. pylori* infection, associated symptoms, recurrence, and complications.

Our study has some limitations. First, our sample size was not large enough and became smaller when groups were subdivided. Randomization of individuals was not performed from the beginning; so during recruitment, only symptomatic and double‐positive (oral and gastric) patients were considered until 50 individuals with periodontal disease and 50 more without periodontal disease were included. Finally, the follow‐up concluding recurrence was conducted only after 16 weeks of treatment.

## 5 Conclusion

This study offers additional evidence that reinforces the convenience of implementing oral hygiene procedures and systemic therapy to increase the eradication of gastric *H. pylori* and reduce recurrence and gastric complications. For the first time, we explored the efficacy of systemic therapy plus oral hygiene (periodontal treatment and/or mouthwash) in double‐positive Mexican patients and demonstrated higher eradication rates and lower recurrence compared with systemic treatment alone or without the use of NEW mouthwash. Thus, the importance of eradicating and controlling the bacteria, not only in gastric tissue but also in the oral cavity, for successful treatment was proven. Our results show that NEW 0.0015%, a non‐xenobiotic substance, is more convenient as an antiseptic mouthwash than chlorhexidine gluconate 0.12% due to its efficacy and safety in the short and long term. Studies with a greater number of individuals and longer follow‐up periods are needed to verify these results.

## Author Contributions

V.H.U.‐B. conducted the research and investigation process, performing the experiments and data collection. B.A.P.‐M. developed the idea and formulated the research, wrote the initial draft. A.N.C.‐P. conducted the patient clinical research and investigation process. J.A.J.‐D.V. conducted the patient clinical research and investigation process. W.J.A.‐F. conducted the patient clinical research and investigation process. N.M.‐S. developed the idea and formulated the research. M.A.R.‐L. developed the idea and formulated the research. M.A.D.L.G.‐R. developed the idea and formulated the research, conducted the research and investigation process, management, and coordination responsibility for the research activity planning and execution. All authors reviewed the manuscript and approved its submission.

## Declaration of Generative AI and AI‐Assisted Technologies in the Writing Process

The authors did not use any generative AI or AI‐assisted technology tool during the preparation of this work.

## Ethics Statement

The Institutional Ethics Committee and the Health Sciences Research and Development Center (CIDICS) of the Autonomous University of Nuevo Leon (UANL) approved the study in January 2020; SPSI‐010613; No. 00208.

## Consent

Written informed consent was obtained from each participant before the study.

## Conflicts of Interest

Brenda Astrid Paz‐Michel, Nicolas Mervitch‐Sigal, and Mario Alfredo Rodríguez‐León are employees at Esteripharma S.A. de C.V. but did not participate in the decision to publish the study results nor in the selection of the volunteers or its development. The other authors declare no conflicts of interest.

## Supporting information

Supporting information.

## Data Availability

The data supporting the study findings are available from the corresponding author upon reasonable request.
