# Supplementary material for: Oral Hygiene With Neutral Electrolyzed Water and Systemic Therapy Increases Gastric Helicobacter pylori Eradication and Reduces Recurrence
Source: Clin Exp Dent Res. 2024 Jul 7;10(4):e927. doi: 10.1002/cre2.927 (PMC11228356; doi:10.1002/cre2.927)
Supplement: Supplementary file 1 — Supporting information. [file CRE2-10-e927-s001.docx]

**Table S1.** Eradication of symptoms associated with *H. pylori* infection 16 weeks after treatment

| **Group** | ***Upper chest pain*** | | | ***Heartburn*** | | | ***Nausea/Vomiting*** | | | ***Early Satiety*** | | |
| --- | --- | --- | --- | --- | --- | --- | --- | --- | --- | --- | --- | --- |
|  | Present | Absent | P-value^†^ | Present | Absent | P-value^†^ | Present | Absent | P-value^†^ | Present | Absent | P-value^†^ |
| **NS + NS-PT [N = 19]** | 9 (47.4%) | 10 (52.6%) | <0.001^***^ | 9 (47.4%) | 10 (52.6%) | 0.001^***^ | 9 (47.4%) | 10 (52.6%) | 0.003^**^ | 10 (52.6%) | 9 (47.4%) | <0.001^***^ |
| **NEW + NEW-PT [N = 45]** | 3 (6.7%) | 42 (93.3%) |  | 4 (8.9%) | 41 (91.1%) |  | 5 (11.1%) | 40 (88.9%) |  | 4 (8.9%) | 41 (91.1%) |  |
| **Total** | 12 (18.8%) | 52 (81.2%) |  | 13 (20.3%) | 51 (79.7%) |  | 14 (21.9%) | 50 (78.1%) |  | 14 (21.9%) | 50 (78.1%) |  |

NS, mouthwash with normal saline; NEW, Neutral Electrolyzed Water; NS-PT, mouthwash with normal saline and periodontal treatment; NEW-PT, mouthwash with NEW and periodontal treatment.

^†^Statistical significance according to Pearson’s Χ^2^ between positive and negative individuals; ^*^P = 0.05, ^**^P = 0.01, ^***^P ≤ 0.001.
